# Lecture on the Diagnosis and Treatment of the Principal Forms of the Paralysis of the Lower Extremities

**Published:** 1861-09

**Authors:** 


### Book Notices.

LECTURES ON THE DIAGNOSIS AND TREATMENT OF THE PRINCIPAL FORMS OF THE PARALYSIS OF THE LOWER EXTREMITIES.—By E. Brown-Séquard, M. D., F. R. S., etc., etc. Philadelphia: J. B. Lippincott & Co. 1861.

This is the title to a neatly printed volume of 118 pages, embracing four lectures on the nature, diagnosis, and treatment of the various forms of paralysis of the lower extremities. It is an interesting and highly instructive volume, and will abundantly repay the practitioner for its purchase and careful perusal.

The author's *pathology* may be stated in very few words. He regards paralysis as arising from two different and essentially opposite conditions of the spinal cord or central parts of the nervous system. The first condition consists in a *contraction* of the blood-vessels, and consequent diminution of the quantity of blood in the part, with diminished nutrition. This condition he regards as capable of arising from irritation in the extremities of any of the sensitive nerves, whether distributed upon internal or external organs; and hence, he gives to cases originating thus, the name of *reflex* paralysis. The same pathological condition, though more fixed and permanent, exists in those cases, accompanied by *white softening* of the cord.

The second condition productive of paraplegia, consists in a dilated or hyperœmic state of the vessels of the spinal cord, and is met with in two stages, namely, congestion and inflammation.

In regard to the mode by which the supposed contraction of blood-vessels is produced, and in turn, gives rise to paralysis, the author says:

"As it is now well established that blood-vessels contract with energy, and sometimes even are seized with a real and prolonged spasm, whether by a direct influence of their motor nerves, or through an excitation, which, from some centripetal or excito-motor nerve, has been reflected upon them by the cerebro-spinal axis, there is no need of showing here, that blood-vessels are just like muscles of animal life, as regards their relation with the nervous system. This being the case, it is extremely easy to understand how a paralysis of the lower extremities, as well as that of any other part of the body, may be produced by reflex action. In three different places, a contraction of blood-vessels may cause paraplegia—1, in the spinal cord; 2, in the motor nerves; 3, in muscles.

We think it will now be considered possible, if not probable, that the production of reflex paraplegia is due to a contraction of blood-vessels and to the insufficiency of nutrition that follows this condition of the vessels."—(See pp. 24 and 25.)

The author having traced paraplegia to two distinct and essentially *opposite* pathological conditions, the diagnosis of one of these conditions from the other becomes a matter of great practical importance in reference to the adoption of a rational course of treatment. Hence, he devotes the greater part of his second lecture to this topic, to a perusal of which we must refer the reader, making room only for the following concluding remarks:

"A *reflex* paraplegia is almost sufficiently characterized by the absence of the special symptoms of an organic disease of the spine or its contents, and the existence of an incomplete paralysis of the lower limbs that has appeared somewhat slowly after a disease of the urinary or genital organs, or of some other abdominal viscus; after an inflammation of the lung or pleura, or after some kind of irritation of a nerve in its trunk or cutaneous ramifications. In a great many cases of reflex paraplegia, we shall find nothing else upon which to ground our diagnosis. But usually, in a short time, a much greater probability of the accuracy of the diagnosis will spring from the correspondence between changes in the degree of paralysis with changes in the visceral disease, or external irritation that is supposed to have produced the paraplegia."

The author regarding reflex paraplegia as originating from diminished circulation and nutrition of the spinal cord, or some of its centres, recommends a treatment which may be embraced in the following propositions: 1st. Remove as far as possible the external irritation or local visceral disease from which the disturbance of the spinal circulation originated. 2d. To give such remedial agents as are capable of increasing the circulation in, and nutrition of nerve structures. Concerning remedies for this purpose, the author says: "We know but one remedy that really deserves confidence—it is strychnine." He says it acts in two ways: "1st, in increasing the amount of blood in the spinal cord; 2d, in acting in a special and direct manner on the tissue of the cord. As regards the first mode of action, we will only state here, that it is a positive fact that the quantity of blood circulating in the spinal cord, is very much increased, and that consequently its nutrition also is increased. As regards the second mode of action, the admirable researches of MM. Martin, Magron and Buisson have established beyond doubt, that even when the spinal cord does not contain any blood, strychnine directly applied upon, or in that organ, increases so much its vital property, that reflex tetanic spasms may be produced. * * * We shall insist, by and by, on the importance of making use of strychnine persistently in almost all cases of paraplegia, in which there is no inflammation or no congestion of the spinal cord or its membranes."

The power of strychnine to increase the amount of blood in the cord, and to exalt its reflex power or susceptibility, thereby rendering it one of the most efficient remedies we possess in the reflex or functional paralysis, renders it, not only inapplicable, but positively injurious in all cases dependent on either congestion or inflammation of that organ. Hence, the author very justly dwells with much emphasis on the necessity of making a careful diagnosis in every case of paraplegia. Our own observation has confirmed this necessity. There are no affections treated more empyrically than the paralytic. By many, the existence of paralysis, either partial or complete, is sufficient to cause an immediate resort to the use of strychnine, electricity and kindred excitants, without any clear idea as to the actual pathological condition on which the paralysis depends.

This work of Dr. Brown-Séquard will do much to bring about a more discriminating practice. It is but a few weeks since we were called to see a gentleman who had nearly lost the use of both lower extremities, with impaired action of the bladder and rectum. His pulse was 85 per minute, and firm under the finger; there was tenderness over the lumbar and sacral vertebræ, and not only tenderness in the part, but pressure over the lower part of the sacrum, would uniformly send a thrill like an electric shock through the lower extremities; there was almost complete loss of sensibility in penis, scrotum and perineum, with occasional, painful, spasmodic action of the muscles of the bladder and rectum, but no involuntary discharges; there was a peculiar sensation, as if a ball or hard substance was constantly pressing on the bottom of the feet towards the toes; so much impairment of motion, that the patient could walk only with great difficulty, yet the sensibility of the extremities instead of being impaired, was rather increased, constituting "*soreness* in the flesh." His urine was scanty and bowels moderately costive, yet his appetite was very good, and there was no general feeling of indisposition. The foregoing symptoms had come on gradually, and though plainly indicating an inflammatory condition of a portion of the spinal cord, yet the patient had been taking strychnine during all the preceding week, and as the paralytic symptoms were increasing, he was about to resort to electricity.

In another case of complete paraplegia, coming under our care in the Mercy Hospital, the paralysis had supervened suddenly, and though accompanied by a quiet pulse, increased heat in the extremities, and such an exalted degree of sensibility, that the slightest touch or motion was painful, yet he had been using as remedal agents, both strychnine and electricity. It is hardly necessary to add, that in both these cases the symptoms increased in severity while the use of the strychnine was continued.

The treatment which our author recommends for paraplegia depending on chronic inflammation or congestion of the spinal cord or its membranes, consists in the avoidance of a dorsal position, the daily use of hot water douches over the spine, dry cupping in the same region, and the internal use of belladonna, ergot of rye, and in those cases accompanied by effusion, the iodide of potassa. In regard to the *modus operandi* of belladonna and ergot, he says:

"Amongst the remedies to be employed internally, the most active are those which have the power of diminishing the congestion of the spinal cord. The two which seem to be most powerful in this respect are *belladonna and ergot of rye.* Experiments upon animals have shown to me, in the most positive manner, that these two remedies are powerful excitants of unstripped muscular fibres, in blood-vessels, in the uterus, in the bowels, etc. * * * * Not only have I seen the diminution in the calibre of blood-vessels of the pia mater of the spinal cord taking place in dogs after they had taken large doses of belladonna or ergot of rye, but I have also ascertained that the reflex power of the spinal cord (most likely as a consequence of the contraction of blood-vessels) becomes very much diminished under the influence of these two remedies, which, in so doing, act just in the opposite way to that of strychnine."

Again he says: "Led by the knowledge of the above facts, we have employed belladonna and ergot of rye in cases of paraplegia due to a simple congestion or a chronic inflammation of the spinal cord and its meninges, and we have obtained a greater success than we had dared to hope for."

We have found so much of interest in this little volume, that our notice has already extended much farther than we intended. We cannot too strongly recommend it to the profession. For several years past, we have not only acted on the supposition, that paralysis might arise from the two opposite pathological conditions of the brain and spinal cord described by M. Brown-Sequard, but also, that cases are occasionally met with from loss of susceptibility in the *muscles* of the paralyzed part, without any morbid condition of the spinal cord or other nerve-centres. And we are not sure, but a number of cases, ranked by the author just named, as cases of reflex paraplegia, are really such as have the pathological lesion primarily in the muscular instead of the nerve structure.

